# The Field of Science

**Published:** 1894-05-19

**Authors:** 


### The Field of Science.

The ninth Exhibition of Hygienic and Food Products takes place in Rome next month. This annual exhibition is organised by the International Association for the Advancement of Hygiene at Brussels. Professor Baccelli, president of the International Medical Congress, has accepted the presidency of the Governing Committee.

Dr. Koch's able Japanese pupil, Kitasato, has certainly received much encouragement from his country. Firstly, the Government provided him with 45,000 dols. to start with, after which he was annually to receive 15,000 dols. Already we hear of the successful manner with which he is treating consumption in Japan, the tuberculin he employs being modified by himself.

Some interesting experiments have recently been tried in America with a view to ascertain what effect, if any, colour has upon certain forms of disease. The test was first applied upon small-pox patients, a number of whom were placed in a room which admitted only of a red light. For the most part these patients were suffering under severe attacks of the malady, half of them being unvaccinated children. The report on this experiment declares that safe and speedy recoveries are registered of all those thus experimented upon, and few scars were left behind on the patients.

Some remarkable and interesting facts have been brought before our notice, consequent on Professor H. M. Ward's researches on the influence of light on bacteria. The rays of the naked arc light, the Professor has successfully demonstrated, have a useful disinfectant action. In hospitals and in railway carriages, he points out, the light has a distinct hygienic value on this account. In his investigations on this head Professor Ward found no perceptible action on the spores of bacteria through the infra-red, yellow, or orange rays, whilst all the spores were injured by the rays of violet and blue. This discovery of the value of electric light as a disinfectant opens up a wide field of speculation.

We learn from internal sources that no cases of enteric fever have occurred at Worthing since the commencement of the year, and that the town now presents a clean bill of health. We might go further, and add a "very" clean bill of health, for as a leading medical journal comments, "a town which, like Worthing, has had its lesson, and put its house in order, is often really in a better sanitary condition than many another which for want of such a salutary warning, is able to trade on its good name, whilst its evil course remains unchecked." M. Hermite's system of treating sewage matter with electrolysed sea water was rejected on account of expense. Some time back we described how the experiments were being conducted at Worthing, with a view to the scheme of the French scientist being applied in assisting the town out of its insanitary difficulties. An abundant supply of fresh water has now been obtained from the village of Broadwater, but the corporation have gone further than this. In the event of the town extending, and to avoid pollution thereby, a site has been selected in the South Downs where two million gallons of pure water can daily be secured.

It appears that the Indian Mohammedan authorities are agreeable to the improvements recommended by the International Cholera Conference in Paris. The information furnished on this head to Mr. E. Hart, prior to his making the proposals which formed the basis of the recent International Conference in Paris, comes, we learn, from a definitely authorised source. The Koran is further brought to bear on the sanitary aspect of these past gatherings of pauper pilgrims, and it is urged as contrary to Koranic prescription that they should thus undertake such journeys without making proper provision for their families who are left behind. In thus prohibiting the massed pauper pilgrimages to the grave of the Prophet, we can look for a greater immunity from the scourge of disease which has yearly followed immediately in the train of the faithful.

It has recently been called attention to in the English press, how the nomenclature of streets in the French capital serves also as a commemorative tribute to science. We find centred round La Salpêtrière Hospital, for instance, the names of various medical men who rose to the front rank in their profession, among which two English physicians, Harvey and Jenner, are conspicuous. Near the Arc de Triomphe occur the names of Kepler, Euler, Newton, and Copernicus, whilst the Jardin des Plantes is appropriately bounded by streets named respectively after Cuvier, Buffon, and Geoffrey St. Hiliare. The above simple facts, it has been commented, show how the municipal authorities of Paris regard the names of men of science, deeming this a small, but significant memorial. Similar authorities on this side the Channel have notyet reached the condition of mind in which scientific workers are thus considered worthy of such recognition.

It is interesting to consider the various methods urged by M. Naquet in his brave endeavour to make a stand against the gradual diminution of the French population. No doubt M. Naquet has fully realised the dimensions of his task, but his late successful propaganda for the divorce laws of his country should give him courage to continue. It has already been suggested that freedom from taxation ought to be observed in the cases of parents with prolific families, and that substantial recompenses in money should further reward these worthy persons. M. Naquet's system, on the other hand, embraces—firstly, rigid sanitary measures in poor districts, and even in country villages where epidemics are frequent and destructive; secondly, increased surveillance of the children sent out to nurse; and, thirdly, greater facilities of naturalisation. The mortality of children confided to the care of "nurses" in the country is declared to be 30 per cent. by the promoter of these improved measures. The French economist suggests that Belgian, Italian, and Swiss aliens might be easily assimilated in the second generation, "if they were helped to forget that they were foreigners." Of course, it depends on what aliens are thus to be naturalised; but somehow M. Naquet's suggestion gives one the idea of "anything" being better than nothing, and that the outcasts of one nation will step in nicely when the home supplies of another fail.

